# Predicting the individualized risk of poor adherence to ART medication among adolescents living with HIV in Uganda: the Suubi+Adherence study

**DOI:** 10.1002/jia2.25756

**Published:** 2021-06-09

**Authors:** Rachel Brathwaite, Fred M Ssewamala, Torsten B Neilands, Moses Okumu, Massy Mutumba, Christopher Damulira, Proscovia Nabunya, Samuel Kizito, Ozge Sensoy Bahar, Claude A Mellins, Mary M McKay

**Affiliations:** ^1^ International Center for Child Health and Development Brown School Washington University in St. Louis St. Louis MO USA; ^2^ Division of Prevention Science Department of Medicine University of California San Francisco CA USA; ^3^ School of Social Work University of Illinois at Urbana‐Champaign Champaign IL USA; ^4^ Department of Health Behavior and Biological Sciences School of Nursing University of Michigan Ann Arbor MI USA; ^5^ International Center for Child Health and Development Masaka Uganda; ^6^ Department of Global Health School of Public Health Boston University Boston MA USA; ^7^ HIV Center for Clinical and Behavioral Studies New York State Psychiatric Institute and Columbia University Medical Center New York NY USA; ^8^ Brown School Washington University in St. Louis St. Louis MO USA

**Keywords:** HIV/AIDS, ART adherence, adolescents, viral load, prediction modelling

## Abstract

**Introduction:**

Achieving optimal adherence to antiretroviral therapy (ART) among adolescents living with HIV (ALWHIV) is challenging, especially in low‐resource settings. To help accurately determine who is at risk of poor adherence, we developed and internally validated models comprising multi‐level factors that can help to predict the individualized risk of poor adherence among ALWHIV in a resource‐limited setting such as Uganda.

**Methods:**

We used data from a sample of 637 ALWHIV in Uganda who participated in a longitudinal study, “Suubi+Adherence” (2012 to 2018). The model was developed using the Least Absolute Shrinkage and Selection Operator (LASSO) penalized regression to select the best subset of multi‐level predictors (individual, household, community or economic‐related factors) of poor adherence in one year’s time using 10‐fold cross‐validation. Seventeen potential predictors included in the model were assessed at 36 months of follow‐up, whereas adherence was assessed at 48 months of follow‐up. Model performance was evaluated using discrimination and calibration measures.

**Results:**

For the model predicting poor adherence, five of the 17 predictors (adherence history, adherence self‐efficacy, family cohesion, child poverty and group assignment) were retained. Its ability to discriminate between individuals with and without poor adherence was acceptable; area under the curve (AUC) = 69.9; 95% CI: 62.7, 72.8. There was no evidence of possible areas of miscalibration (test statistic = 1.20; *p* = 0.273). The overall performance of the model was good.

**Conclusions:**

Our findings support prediction modelling as a useful tool that can be leveraged to improve outcomes across the HIV care continuum. Utilizing information from multiple sources, the risk prediction score tool applied here can be refined further with the ultimate goal of being used in a screening tool by practitioners working with ALWHIV. Specifically, the tool could help identify and provide early interventions to adolescents at the highest risk of poor adherence and/or viral non‐suppression. However, further fine‐tuning and external validation may be required before wide‐scale implementation.

## 1 Introduction

Persons living with HIV must initiate and consistently adhere to antiretroviral therapy (ART) to ensure HIV viral suppression [1], avoid drug resistance [2, 3], and maintain adequate immune functioning thereby improving quality of life [4] and reducing the possibility of death from AIDS [3]. Viral suppression reduces the risk of sexually transmitting the virus to others through condomless sex [5, 6]. UNAIDS’ 95‐95‐95 treatment targets [7] highlight the importance of adherence in preventing new infections, reducing mortality and ending the HIV epidemic by 2030 [6].

Global trends show that over the last decade, AIDS‐related deaths have increased among adolescents living with HIV (ALWHIV) compared to older age groups, partly due to delayed treatment initiation and poor treatment adherence [8, 9]. Sub‐Saharan Africa (SSA), a resource‐constrained region, bears the highest global burden of ALWHIV (89%) [8], with two‐thirds of daily global infections occurring among youths aged 15 to 24 years [10]. ALWHIV in SSA are a vulnerable group at a higher risk of non/poor adherence than adults [11]. In Uganda, a SSA country with high HIV prevalence, viral suppression among youths aged 15 to 24 is 44.9% among females and 32.5% among males [12].

We utilize two theoretical frameworks, Asset theory [13] and the Social Ecological Model (SEM) [14], to guide our understanding of the multi‐level factors that may influence ART adherence among ALWHIV in poor communities [11, 15, 16, 17]. Asset theory posits that if individuals have access to tangible assets, including monetary savings, this can positively alter their thinking, attitudes and subsequent behaviour [13]. Having a greater sense of economic safety and security about the future produces feelings of optimism and improved psychological functioning. Therefore, improving the financial situation of ALWHIV can increase their access to opportunities (e.g. food security and transport affordability to healthcare facilities), which in turn may positively impact their behaviour (e.g. adherence to medication) [17, 18, 19, 20].

SEM [14] posits that ART adherence among ALWHIV is influenced by intersecting multi‐level factors that span across individual, household and community/structural levels [16, 21]. In this paper, the Asset theory provides a nuanced understanding of economic factors nested within the SEM’s structural level.

Unlike previous studies among ALWHIV that primarily focused on utilizing traditional regression methods (which adjust for covariates) to investigate and identify statistically significant associations between risk factors and adherence at the population level, we utilize multi‐level factors to predict the individualized risk of poor adherence in unseen cases [22, 23]. We utilize prediction modelling techniques [24] to (1) identify a model comprising a combination of a subset of multi‐level factors that best predicts individualized risk of ART adherence among ALWHIV; (2) evaluate the extent to which the derived model can discriminate between poor and optimal adherence among ALWHIV and (3) assess the agreement between model predicted and actual adherence among ALWHIV (calibration). Before being implemented in the field, this model will be subjected to additional studies to improve its predictive values and accuracy, and undergo external validation among ALWHIV populations in other settings.

## 2 methods

### 2.1 Study setting and study participants

We used data from a six‐year longitudinal study called Suubi+Adherence (2012 to 2018 NCE), conducted in 39 health clinics across six geographical districts (Masaka, Kalungu, Lwengo, Rakai, Kyotera and Bukomansimbi) in Southwestern Uganda [20]. Only adolescents who voluntarily provided assent and informed caregiver consent were allowed to participate. A total of 702 ALWHIV were enrolled. Eligibility included: (1) medically diagnosed with HIV and aware of their HIV status; (2) living within a family (could be biological family or caregiver, but not an institution); (3) aged 10 to 16 at baseline; (4) prescribed ART medication and (5) receiving HIV care and treatment at one of 39 clinics enrolled. Participants were randomized at the clinic level to either a family economic intervention arm or a control arm receiving usual care. Assessments were conducted at baseline, 12, 24, 36 and 48 months. Details on the intervention are provided elsewhere [20]. Briefly, participants in the control arm received bolstered standard of care (BSOC), which involved medical and psychosocial support. The intervention arm received BSOC and child development savings accounts for long‐term saving goals, training on financial management, starting a business and mentorship. For this analysis, we included participants who were followed up at all time points (n = 656) and had complete data on predictors at 36 months and the outcome at 48 months (n = 637).

### 2.2 Measures of ART adherence

Adherence at 48 months was assessed using self‐reported responses and viral load. Viral load was categorized into a binary indicator (detectable vs. undetectable) using cutoffs established by the WHO, with levels exceeding 1000 HIV RNA copies/mL as detectable [25]. Additionally, participants were asked “*In the last 30 days, on how many days did you miss at least one dose of your HIV medications*?” We utilized a conservative measure of adherence in order to capture all potential cases of non‐adherence and participants who selected missing two or more days in the last 30 days were categorized as having “poor adherence.” We deliberately included a self‐reported measure of adherence in the analysis because in many low‐resource settings (including Uganda) frequent viral load testing may be limited due to lack of resources, hence self‐reports can provide low‐cost alternative measures of adherence [26]. We sought to predict poor adherence or viral non‐suppression since either state is considered a poor HIV health outcome and would benefit from intervention. For brevity, we refer to this outcome as “ART adherence.”

### 2.3 Predictors of ART adherence

Predictors encompassed factors previously associated with ART adherence among ALWHIV [27]. These included: (1) demographic factors, that is age [28], gender [18]; (2) individual‐level psychosocial and HIV‐related factors, that is depression, hopelessness [29, 30], adherence self‐efficacy [29], substance use [31], HIV disclosure [32] and history of ART adherence [33]; (3) household‐level factors, that is family cohesion [34], biological caregiver [28], HIV treatment supporter [28]; (4) community/structural‐level factors, that is social support network [34], distance to health facility [18], HIV‐related stigma [35]; asset ownership [18], child poverty [28], economic intervention assignment [36]. Though the intervention was effective in the parent study [36], in this study the outcome is different. In the parent study, detectable viral load, defined as >40 copies/mL was used as the outcome to evaluate intervention efficacy. In contrast, we used a combination of detectable viral load (>1000 copies/mL) and poor self‐reported adherence. We revisited our data and confirmed that there is not a statistically significant difference between the study arms on the composite viral load and adherence outcome used in this manuscript. Therefore, we combined the arms, and used the intervention as a predictor. To address temporality, predictors were measured at 36 months to determine factors that predicted poor 48‐month ART adherence. Refer to Table S2.

### 2.4 Ethics

The Suubi+Adherence study received IRB approval from Columbia University, from the Uganda National Council for Science and Technology, and the Makerere University School of Public Health Higher Degrees Research Ethics Committee (210). The trial was registered at ClinicalTrials.gov, registration number NCT01790373.

### 2.5 Statistical analysis

Analyses were conducted in STATA Version 16.1. Sample characteristics and distributions of categorical predictors were summarized using numbers and percentages, and using median and interquartile ranges (IQR) for continuous variables. The least absolute shrinkage and selection operator (LASSO) penalized regression was used to select the best subset of predictors of poor adherence. LASSO selects a subset of predictors by shrinking the coefficients of the least contributive variables to zero, thereby excluding them from the model. The tuning parameter lambda, which determines the amount of coefficient shrinkage, was selected by 10‐fold cross‐validation [37]. We also used 10‐fold cross‐validation to generate a realistic estimation of the predictive performance (area under the curve (AUC)) of the final model in new cases (an important goal of prediction modelling). Ten‐fold cross‐validation is preferable to split sample validation since it prevents obtaining overly optimistic estimates of predictive performance in unseen cases [38]. The value of the AUC indicates the ability of the model to differentiate between ALWHIV with poor and optimal adherence, with [39] thresholds of discriminative quality ranging from 50% indicating inability to discriminate between individuals with the outcome or not; 70% to 80% – acceptable; 80% to 90% – excellent; >90% – outstanding performance [40]. Bootstrapped Bias‐corrected (BC) 95% confidence intervals for the AUC were also generated. The agreement between model‐predicted risks and actual observed rates of poor adherence was evaluated via calibration belt plots which allowed visual inspection of where observed frequencies significantly differed from expected probabilities and the localized direction of deviation (miscalibration) at certain confidence levels [41]. A calibration test assessed whether any deviations from the bisector [45° line of perfect fit] were significant, was also included in the calibration belt plot [42, 43]. To assess overall performance, we used the Brier score, which is a measure of disagreement between the forecasted predictions and the observed outcome, and which carries benchmark values of 0 for no disagreement, 1 for complete disagreement and 0.25 for predictions no better than chance (50/50) [44].

### 2.6 Sensitivity analyses

As a sensitivity analysis, we examined whether the clustered nature of the data affected its discrimination by re‐estimating the AUC using bootstrap‐resampling to account for clustering by clinics. We also performed a sensitivity analysis to evaluate whether including cases with incomplete data in the analysis would affect the model results. We used the expectation‐maximization (EM) algorithm [45, 46] to impute missing values and refitted the predictive model to the imputed data using the same lasso method and evaluated model performance using AUC and calibration statistics as described above for the main model.

## 3 Results

### 3.1 Sample characteristics

The mean (SD) age at 36 months of follow‐up was 15.3 (2.2) years. Age ranged from 13 to 20 years, the majority (83.5%) were aged 13 to 17 years, and 56% were females (Table 1).

**Table 1:** Distribution of predictors (assessed at 36 months) in the ALWHIV sample

	Potential predictors included in the model	Categories and coding for categorical variables and description of continuous variables	Total N = 637 n (%)/[Range]^b^/Median (IQR)/α
Demographic factors			
1	Age group	13 to 17 years	
		18 to 20 years	105 (16.5)
2	Gender	Male = 1	281 (44.1)
		Female = 2	356 (55.9)
Individual level factors			
Behavioural			
3	Substance use	No = 0	621 (97.5)
		Yes = 1	16 (2.5)
4	History of ART adherence	Good = Missed only 1 day or less in the last 30 days during baseline to 36 months of follow‐up = 0	404 (63.4)
		Poor = missed 2 or more days in last 30 days during baseline to 36 months of follow‐up = 1	233 (36.6)
Psychosocial			
5	Depression	Higher scores represent greater depression	[2 to 17] 5 (4, 8) α = 0.64
6	Hopelessness	Higher scores represent greater hopelessness	[2 to 16] 5(3, 7) α = 0.76
7	Adherence Self‐efficacy	Higher scores indicate higher levels of confidence in taking ART medication	[12 to 120] 94 (78, 109) α = 0.85
8	HIV disclosure	None or uncertain = 1	292 (45.8)
		Few, some, or all = 2	345 (54.2)
Household level factors			
9	ART treatment supporter	Yes = 1	492 (77.2)
		No = 2	145 (22.8)
10	Family cohesion	Higher scores represent greater family cohesion.	[6 to 30] 23 (18, 27) α = 0.77
11	Caregiver type	Biological caregiver = 1	310 (48.7)
		Non‐biological caregiver = 0	327 (51.3)
Community/structural level factors			
12	HIV‐related stigma	Higher scores represent higher levels of internalized and anticipated stigma	[9 to 36] 17 (13, 21) α = 0.74
13	Social support network^a^	Higher scores represent greater social support.	[21 to 60] 45 (39, 50) α = 0.68
14	Distance to health facility	Very near/near = 0;	461 (72.4)
		Very far/far/don’t know = 1	176 (27.6)
Economic level factors			
15	Asset ownership	High possession [≥7 assets] = 0	549 (86.2)
		Low possession [<7 assets] = 1	88 (13.8)
16	Child poverty	Lower scores representing greater levels of poverty	[0 to 9] 4 (3,5)
17	Economic Group assignment	Control = 1	314 (49.3)
		Intervention = 2	323 (50.7)

^a^ Social support network included from parents and friends only. Social support from classmates and teachers was not assessed since many participants were not in school at the time. α‐Cronbach’s alpha in the sample. IQR refer to interquartile range

^b^ This is the range in the data. Depression measured using the 14‐item version of the Children Depression Inventory (CDI); Hopelessness assessed using 20‐item Beck Hopelessness Scale; Adherence self‐efficacy assessed using the 12‐item HIV treatment adherence Self‐Efficacy Scale; HIV‐Disclosure assessed from the question “Do any of your friends know you are HIV positive?”; ART treatment supporter assessed from the question “Do you have someone to remind you to take ART medication?”; Family cohesion: sum of 6 items from the family environment scale; HIV‐related stigma‐ assessed using the 9‐items from the adapted Berger Stigma scale; Social support network assessed from 12 items adapted from the Social Support Behaviors Scale (friends, parents and guardians); Distance to health facility assessed from the question “How far from your home is the hospital or clinic?”; Asset ownership: evaluation of family ownership of 20 selected assets; Child poverty – 6‐item scale; Economic group assignment – Group assigned in RCT at baseline.

### 3.2 Relationship between self‐reported adherence and viral suppression

Adolescents who self‐reported poor adherence at 48 months (missed 2 or more days in the last 30 days approximately <95% adherence rate) had 1.82 (95% CI: 1.02, 2.99) times the odds of a detectable viral load at 48 months compared to those who missed one day or less, after standard errors were adjusted for clustering by clinics (Refer to Table S1). Adolescents who missed two or more days during baseline measure to 36 months of follow‐up had 2.41 (95% CI: 1.52, 3.79) times the odds of detectable viral load at 48 months, compared to those missing one day or less. These positive associations indicate that in the absence of a viral load, self‐reported adherence could be a reliable indicator of true adherence and, ultimately viral suppression. Therefore, in this analysis, participants with detectable viral load or those who missed two or more days in the last 30 days were considered as having “poor adherence.”

### 3.3 Prevalence of poor adherence and predictors retained

The prevalence of “poor adherence” at 48 months among ALWHIV was 29.0% (95% CI: 25.6, 32.7; n = 185/637). Separately, the prevalence of self‐reported non‐adherence was 16.6%, and viral non‐suppression was 17.4%. Following Lasso regression, and based on a mean lambda of 0.0140954, five out of the 17 predictors were retained in the model for predicting which ALWHIV will have poor adherence at 48 months (Table 2). The five predictors retained included individual (adherence self‐efficacy score, history of ART adherence), household (family cohesion) and economic (child poverty and economic group assignment) factors (Table 2).

**Table 2:** Unstandardized penalized regression coefficients of predictors retained in the lasso model to predict poor adherence

	Predictors	Model derived using lasso regression (N = 637)
	Intercept	−0.1073331
	Demographic factors	
1	Age group	
	13 to 17	x
	18 to 20	x
2	Gender	x
	Individual level factors	
	Behavioural	
3	Substance use	
	Never used drugs	x
	Used drugs	x
4	History of ART adherence	
	Good adherence	−1.149057
	Poor adherence	x
	Psychosocial	
5	Depression	x
6	Hopelessness	x
7	Adherence self‐efficacy	−0.0002111
8	HIV disclosure	
	None or uncertain	x
	Few/some/all	x
	Household level factors	
9	ART treatment supporter	
	Yes	x
	No	x
10	Family cohesion	0.0080239
11	Caregiver type	x
	Community/structural level factors	
12	HIV‐related stigma	x
13	Social support network	x
14	Distance to health facility	
	Very near or near	x
	Very far, far, missing, n/a, don’t know	x
	Economic level factors	
15	Asset ownership	
	High possession	x
	Low possession	x
16	Child poverty	−0.0779142
17	Economic intervention group assignment	
	Control group	0.1162283
	Intervention group	x
	Total number of predictors retained from the 17 in the model	5
	AUC (Bootstrap corrected 95% CI) derived using 10‐fold cross‐validation	69.9 (62.7, 72.8)
	AUC (95% CI) derived using 1000 bootstrap resampling which adjusted for clustering by clinics	69.4 (64.7, 73.3)
	Brier score	0.1868

‘x’, excluded from final prediction model after lasso regression. AUC, area under the curve. 95% CI, 95% confidence interval. NB, penalized regression coefficients were derived after a penalty was applied which reduces overfitting of the data during model development. The penalized coefficients are not reflective of true population‐level associations, since these are biased, and so attention should not be placed on interpreting individual predictor coefficients, but on how the model performs with the combination of all predictors together. Using the coefficients from the predictors retained in the model, the probability of poor adherence for an ALWHIV is equal to the inverse of a logistic regression equation as follows: 1/(1 + exp (−(−0.107 − 1.149 × good previous adherence − 0.116 × control group − 0.078 × child poverty score + 0.008 × family cohesion − 0.0002 × adherence self‐efficacy score))).

### 3.4 Discrimination and distribution of risk scores

**Figure 1:**
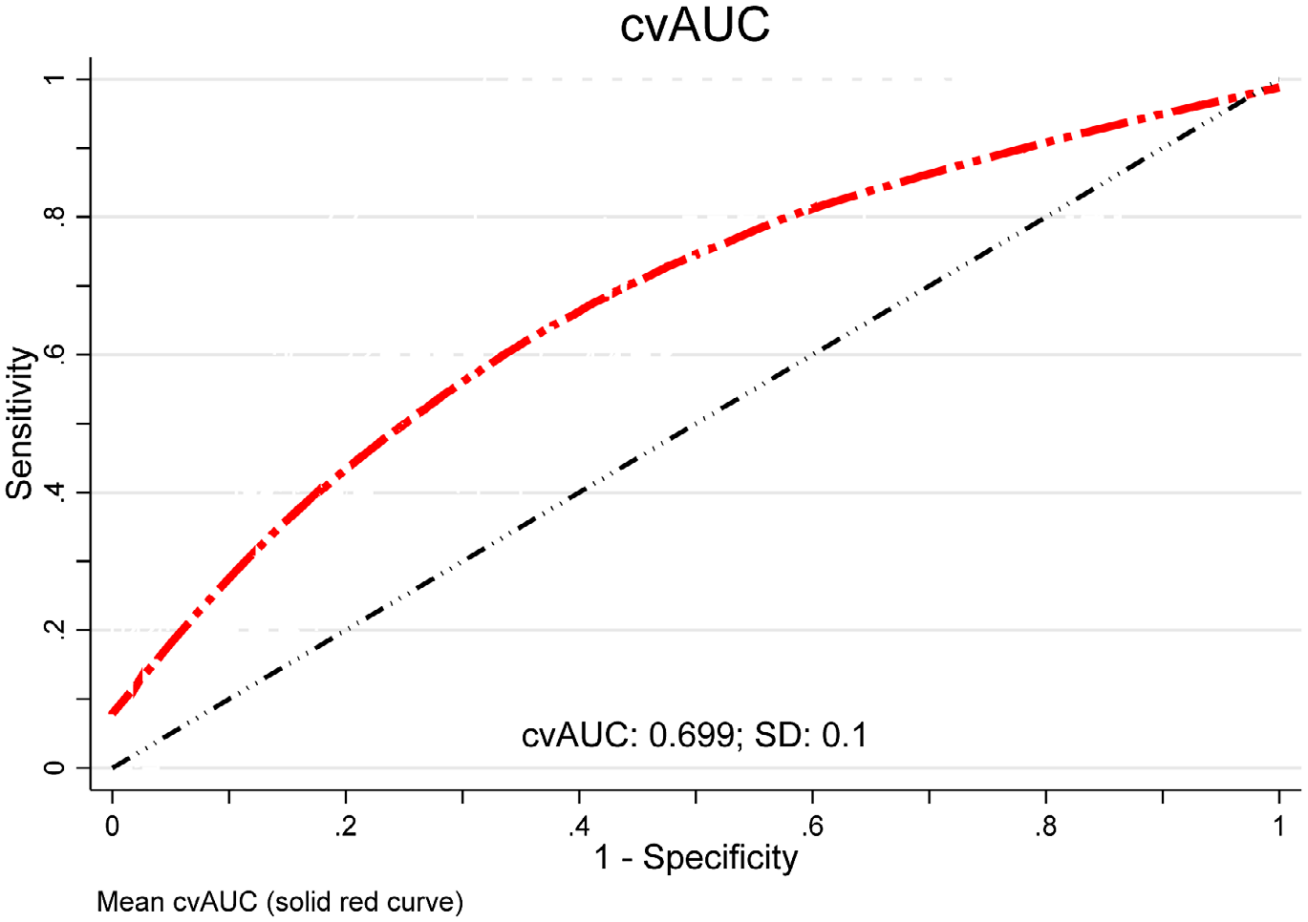
Model discrimination: ROC curve showing mean cross‐validated area under the curve (AUC) resulting after 10‐fold‐cross validation. AUC = 69.9; Bootstrap bias corrected 95% CI 62.7, 72.8. The diagonal line represents a model that discriminates by chance (AUC = 50); the x‐axis shows the proportion without poor adherence who were incorrectly classified as having poor adherence (false positive rate or 1‐Specificity); the y‐axis shows the proportion with poor adherence that were correctly classified as having poor adherence (Sensitivity or true positive rate). CvAUC, mean cross‐validated area under the curve.

**Figure 2:**
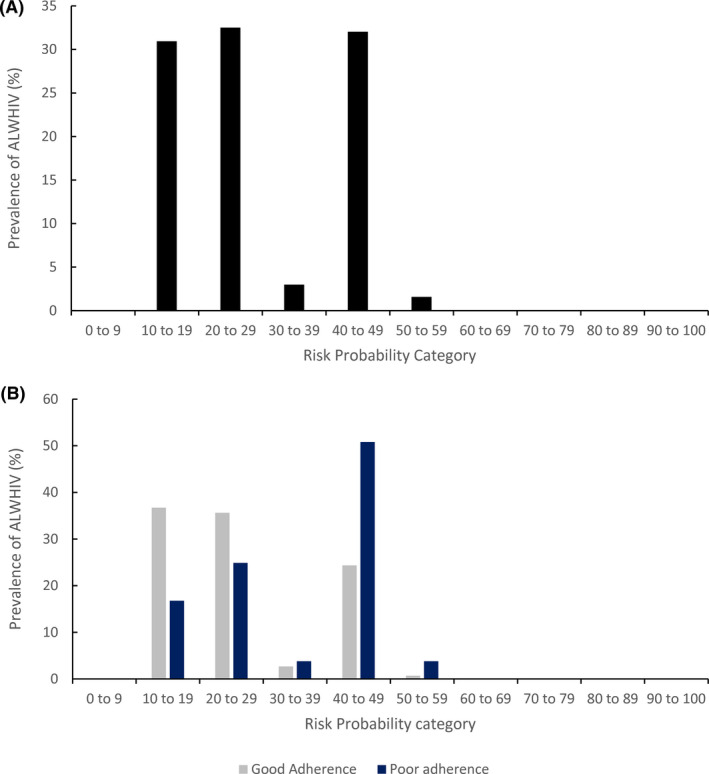
**(A)** Distribution of model predicted risk scores for model derived using lasso regression for predicting poor ART adherence among ALWHIV sample (N = 637). **(B)** Distribution of model predicted risk scores among ALWHIV with poor and good adherence (N = 637).

Following 10‐fold cross‐validation to assess internal validation, the cross‐validated mean AUC for the final model (Figure 1) was 69.9 (BC 95% CI: 62.7, 72.8). This meant that for a randomly selected ALWHIV, there was a 70% probability that the model will correctly assign a higher risk score for an ALWHIV with poor adherence than an ALWHIV with good adherence. The model predicted risk scores for the ALWHIV sample ranged from 12.7% to 51.9%. The median predicted risk score among the sample was 21.9% (IQR: 19.5, 43.0). The distribution of predicted risk scores among the entire sample and among ALWHIV with poor and good adherence is shown in Figure 2a,b. There was a higher prevalence of ALWHIV with poor adherence compared to good adherence for predicted risk scores of 30% and above.

### 3.5 Calibration

**Figure 3:**
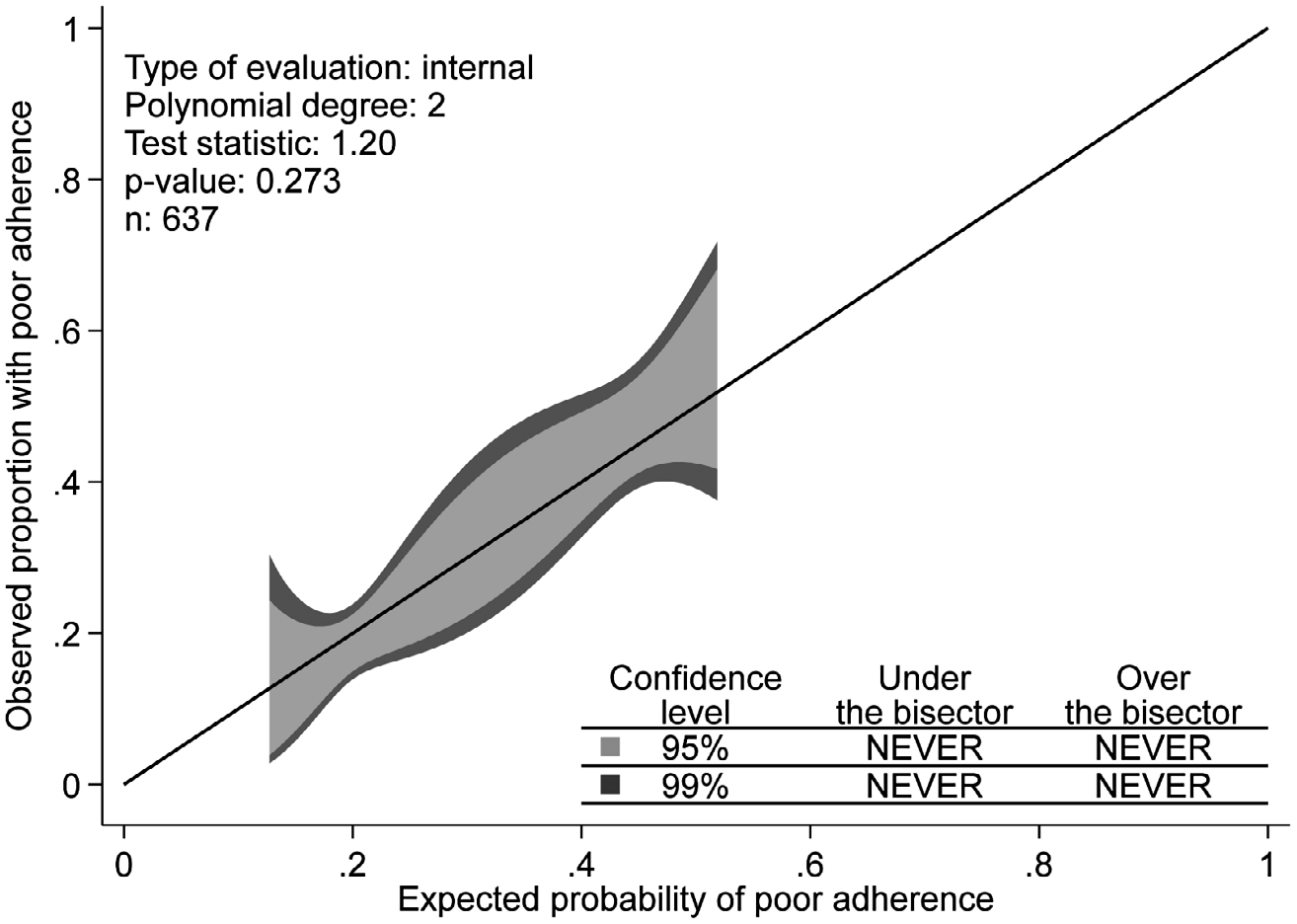
Calibration belt showing deviations from the bisector (45% line of perfect fit) at the 95% (inner belt: light grey area) and 99% (outer belt: dark grey area) confidence levels.

The calibration belt plot indicated that there were no ranges of significant miscalibration at the 95% and 99% confidence levels. The probabilities estimated from the predictive model appeared to match the observed outcome rate across all predicted probabilities at the 95% (inner belt: a light grey area) and 99% (outer belt: a dark grey area) confidence levels (Figure 3). The corresponding *p*‐value was not significant (test statistic = 1.20; *p* = 0.273), indicating no evidence of possible areas of miscalibration. The 95% confidence band’s edges always included the bisector (45° line of perfect fit) suggesting there is no predicted probability significantly different from the observed outcome rate at the 0.05 level (also true for the 0.01 level).

### 3.6 Sensitivity analysis findings

In sum, not much difference in model discriminations was observed when AUCs were derived using bootstrap‐resampling, which accounted for clustering by clinics (Table 2). Hence, the clustered nature of the data did not significantly affect its predictive accuracy (AUC = 69.4; BC 95% [64.7, 73.3]). For the model in which we imputed missing values, the AUC was acceptable (AUC = 70.2 [66.3, 73.8]) (Table S3) but there was some evidence of miscalibration (Refer to Figure S1). Five predictors (adherence self‐efficacy score, history of ART adherence, family cohesion, child poverty and economic group assignment) were retained as important predictors in the model using complete cases, and the same predictors were included in the sensitivity analyses accounting for missing data. Suggesting that, despite missing data, these predictors were consistently important for predicting poor adherence among ALWHIV in this setting. Age and primary caregiver were retained as additional predictors in the sensitivity analysis.

## 4 Discussion

Our findings illustrate that predicting the individualized risk of having detectable viral load and poor 30‐day adherence was possible using a combination of individual (history of ART adherence, self‐efficacy), household (family cohesion) and structural (child poverty and intervention assignment) risk factors. The 95% CI (62.7 to 72.8) indicates that the true discriminative ability (AUC) of the model is acceptable. There was no evidence of miscalibration, and the model had a good overall performance. Therefore, our analysis offers a promising approach as a screening tool to determine the individualized risk of poor ART adherence among ALWHIV.

The resulting individualized risk score derived from the model may allow practitioners in the health and social work fields working with ALWHIV to flag those with the highest predicted risk, enabling tailoring of adherence‐promoting interventions. Objective measures of ART adherence including real‐time electronic monitors (e.g. wise pills and Medication Event Monitoring System (MEMS) cap) are costly and impractical for long‐term use [47], relying on cheaper and tested self‐reported measures as indicators of adherence in low‐resource communities could be advantageous since these were positively associated with viral load [47, 48]. Knowing ALWHIV’s individualized risk of poor ART adherence in advance is an important step in improving ART adherence. This is a promising step closer to ending the HIV epidemic, especially given strong data that transmission is unlikely in the context of controlled virus, which is the basis for UNAIDS message of Undetectable = Untransmittable [49].

Our understanding of poor/non‐adherence among ALWHIV is based on population‐level analyses – which are necessary to understand population‐level trends and sub‐group differences. However, individualized risk assessment approaches have the potential to advance our understanding, specifically in identifying adolescents at risk for poor health outcomes even before these outcomes occur. Clinic medical records have the potential to advance individualized risk assessments, even in low‐resourced settings such as SSA. Supported by The President’s Emergency Plan For AIDS Relief (PEPFAR) and the Global Fund, many HIV clinics in SSA have adopted electronic medical record systems that provide a wealth of clinical and psychosocial information about HIV patients, including ALWHIV. However, these systems are vastly under‐utilized to inform clinical decision making, probably because these systems have been envisioned as data repositories rather than systems that health workers can deploy to inform clinical decision making. Given the multitude of predictors associated with poor adherence, the first step is to identify potential clinically relevant predictors (including their relative predictive value) to inform individualized assessments for the risk of non‐adherence. Based on pre‐established evidence‐based criteria, health workers and public health specialists can identify at‐risk adolescents, and also develop and/or implement interventions to mitigate this risk. As such, our study is the first step towards identifying clinically relevant factors that can facilitate individualized approaches. Given that this study relies on validated self‐report instruments, these questionnaires can be transformed into self‐report assessments administered periodically to inform individualized risk assessments. The increasing availability of low‐cost technologies such as tablet computers, Audio Computer‐Assisted Self‐Interview (ACASI) software and the internet all provide new frontiers for integrating such assessments into routine patient care, even in low‐resourced settings.

These findings should be interpreted in consideration of the study limitations. Our study examined a set of predictors based on available data. Still, based on the AUC, this model’s predictive value could be improved by adding other factors such as regimen type and related information from the health system level. Although the AUC of 70% may be acceptable for the development of an initial screening tool, there is a need for further expansion and validation work to optimize its accuracy before deployment in a clinical setting to improve clinical decisions. We were unable to evaluate the transportability of the model to ALWHIV in different settings via external validation. We acknowledge that our model development sample comprised participants in a randomized controlled trial, 50.7% of whom received a combination economic intervention. The unique components of this intervention may not be available or easily replicated in other settings. Thus, this predictor (economic intervention group assignment) may be missing during external validation in other ALWHIV samples in SSA, possibly limiting generalizability. The findings may also not be generalizable to more marginalized ALWHIV populations in SSA, including persons involved in sex work and people who inject drugs, who are disproportionately affected by HIV, have higher non‐adherence rates, and thus may have different risk factors for adherence and viral suppression than what was considered [50].

We utilized a conservative approach to measuring adherence although a lower adherence threshold may be required to achieve viral suppression with newer‐generation antiretroviral medications which are more forgiving if more doses are missed [1, 51]. Since we were unaware of which antiretroviral medication these ALWHIV were taking, we used the more conservative measure. Moreover, very few participants in our sample reported non‐adherence below 95%, necessitating the use of the 95% cutoff in this study. Future studies should extend this work in samples with subpar adherence. Our findings may only be generalizable to ALWHIV, who live within a family setting and are aware of their HIV status. This strengthens the need for external validation and substituting predictors unavailable for the current analyses with context‐specific predictors from the new setting during the model updating and external validation processes [52]. The clinical utility of the score and feasibility and acceptability of this tool also needs to be measured before being used in practice. Although a factor may be statistically significantly associated with poor adherence, as seen in previous research [18, 28], it may not necessarily have predictive power [53], and not contribute to predicting poor adherence in new cases as was indicated by the subset of predictors excluded after lasso regression. Although sex was not retained as a predictor in the final model, future studies should also consider stratification by sex or exploring interactions between sex and other predictor variables since in SSA, adolescent girls are a vulnerable population disproportionately affected by HIV [54]. Due to our complete case analysis, there may be a potential for selection bias since those lost to follow‐up may be more likely to have a detectable viral load and more at risk of poor adherence. It is possible that some adolescents with detectable viral load who reported complete adherence may have had drug resistance; therefore, there is a need for studies to further our understanding of drug resistance among ALWHIV.

Despite these limitations, in our main analyses, there were 185 participants with the outcome and 17 potential predictors which conforms to the minimum of 10 events per predictor variable (EPV) required for reliable prediction modelling [55, 56]. We also utilized a robust 10‐fold cross‐validation for internal validation, which produces less biased estimates than split‐sample validation, resulting in more confident predictions [38]. The longitudinal design of the data ensured temporal precedence. Advanced knowledge about each adolescent’s individualized risk of poor adherence is a great advantage, as it allows early intervention where possible.

## 5 CONCLUSIONS

The findings support the use of prediction modelling techniques as a useful method that can be leveraged in the fight to end the HIV epidemic. By drawing upon information from multiple sources, a risk score can be derived for each ALWHIV, predicting their risk of poor future adherence. This risk score can be used as a screening tool by practitioners in social and health fields working with ALWHIV to identify and provide interventions to those at the highest risk. However, further fine‐tuning and external validation are required before implementation.

## Competing interest

The authors declare that they have no competing interests.

## Authors’ contributions

R.B. performed the analysis and wrote the first draft of the paper. T.B.N. is the lead statistician on the Suubi+Adherence study, provided overall supervision and guidance of statistical analysis and contributed to revisions of the manuscript. F.M.S. conceptualized the Suubi+Adherence study on which this manuscript is based, was the Lead PI for the study, and supervised the overall writing of the original manuscript. C.D. was the field data manager for Suubi+Adherence Study. C.M. and M.M.M. contributed to the design of the original study and the measures collected. T.B.N., M.O., M.M., S.K., O.S.B., P.N., F.M.S, C.M and M.M.M. contributed to revisions of the manuscript. All authors have read and approved the final manuscript.

## Supporting information


**Table S1**. Logistic regression showing association between self‐reported adherence and viral load
**Table S2**. Description of predictors included in the model and distribution of predictors in the ALWHIV sample
**Table S3**. Unstandardized penalized regression coefficients of predictors retained in the lasso model to predict poor adherence
**Figure S1**. Calibration belt showing deviations from the bisector (45% line of perfect fit) at the 95% (inner belt: light grey area) and 99% (outer belt: dark grey area) confidence levels for the model in which all missing values were imputed.
