# Supplementary material for: Predicting the individualized risk of poor adherence to ART medication among adolescents living with HIV in Uganda: the Suubi+Adherence study
Source: J Int AIDS Soc. 2021 Jun 9;24(6):e25756. doi: 10.1002/jia2.25756 (PMC8188571; doi:10.1002/jia2.25756)
Supplement: Supplementary file 1 — Table S1. Logistic regression showing association between self‐reported adherence and viral load Table S2. Description of predictors included in the model and distribution of predictors in the ALWHIV sample Table S3. Unstandardized penalized regression coefficients of predictors retained in the lasso model to predict poor adherence Figure S1. Calibration belt showing deviations from the bisector (45% line of perfect fit) at the 95% (inner belt: light grey area) and 99% (outer belt: dark grey area) confidence levels for the model in which all missing values were imputed. [file JIA2-24-e25756-s001.docx]

**Predicting the individualized risk of poor adherence to ART medication among adolescents living with HIV in Uganda: The Suubi+Adherence Study**

**SUPPLEMETARY FILE**

**Table S1. Logistic regression showing association between self-reported adherence and viral load**

|  | **Undetectable viral load at 48 months** | **Detectable viral load at 48 months** | |
| --- | --- | --- | --- |
|  | **n (%)** | **n (%)** | **OR (95% CI)**^†^ |
| Missed 1 or less days in last 30 days at 48 months (N=518) | 434 (83.8) | 84 (16.2) | 1.00 |
| Missed 2 or more days in last 30 days at 48 months (N=100) | 74 (74.0) | 26 (26.0) | 1.81 (1.10, 2.99) |
| Good self-reported history^ǂ^ of adherence (N=386) | 337 (87.3) | 49 (12.7) | 1.00 |
| Poor self-reported history^ǂ^ of adherence (N=220) | 163 (74.1) | 57 (25.9) | 2.41 (1.52, 3.79) |

OR-Odds ratios; CI-confidence intervals; ^†^standard errors were adjusted for clustering by 39 clinics; 1.00-reference category; ^ǂ^history-at any time from baseline to 36 months follow-up

**Table S2. Description of predictors included in the model and distribution of predictors in the ALWHIV sample**

|  | **PREDICTORS** | **How defined** | **Categories and coding** | **Total N=637**  **n(%)/ [Range]/ Median (IQR)/ α** |
| --- | --- | --- | --- | --- |
|  | **DEMOGRAPHIC FACTORS** | | | |
| 1 | Age group | Self-reported age at 36 months | 13-17 years | 532 (83.5) |
|  |  |  | 18-20 years | 105 (16.5) |
| 2 | Gender | Self-reported gender at baseline | male=1 | 281 (44.1) |
|  |  |  | female=2 | 356 (55.9) |
|  | **INDIVIDUAL LEVEL FACTORS** | | | |
|  | **Behavioral** |  |  |  |
| 3 | Substance use | Self-reported use of alcohol, cigarettes, or marijuana at 36 months | no=0 | 621 (97.5) |
|  |  |  | yes=1 | 16 (2.5) |
| 4 | History of ART adherence | Self-reported responses to number of days on which missed at least one dose of HIV medication in the last 30 days. Responses from assessments at baseline, 12, 24 and 36 months were combined to determine history of ART adherence. | Good history of adherence=Missed only 1 day or less in the last 30 days at any time during baseline to 36 months follow-up= 0 | 404 (63.4) |
|  |  |  | Poor history of adherence= missed 2 or more days in last 30 days at any time during baseline to 36 months follow-up =1 | 233 (36.6) |
|  | **Psychosocial** |  |  |  |
| 5 | Depression | Assessment of depression at 36 months using the 14-item version of the Children Depression Inventory (CDI) (1). | Higher scores represent greater depression | [2-17]  5 (4, 8)  α=0.64 |
| 6 | Hopelessness | Assessment of hopelessness at 36 months using 20-item Beck Hopelessness Scale (BHS) (2). | Higher scores represent greater hopelessness | [2-16]  5(3,7)  α=0.76 |
| 7 | Adherence Self-efficacy | 12-item HIV treatment adherence Self-Efficacy Scale to measure adherence self-efficacy at 36 months(3) | Higher scores indicate higher levels of confidence in taking ART medication | [12-120]  95 (79, 109)  α=0.85 |
| 8 | HIV disclosure | Do any of your friends know you are HIV positive? | None or uncertain | 292 (45.8) |
|  |  |  | Few, some, or all | 345 (54.2) |
|  | **HOUSEHOLD LEVEL FACTORS** | | | |
| 9 | ART treatment supporter | Someone to remind them to take ART medication at 36 months | Yes=1 | 492 (77.2) |
|  |  |  | No=2 | 145 (22.8) |
|  |  |  |  |  |
| 10 | Family cohesion | Sum of 6 items from the family environment scale at 36 months (4) | higher scores represent greater family cohesion. | [6-30]  23 (18, 27)  α=0.77 |
| 11 | Caregiver type | Type of caregiver at 48 months | Biological caregiver=1 | 310 (48.7) |
|  |  |  | Non-biological caregiver=0 | 327 (51.3) |
|  | **COMMUNITY/STRUCTURAL LEVEL FACTORS** | | | |
| 12 | HIV-related stigma | Sum of 9 items from the adapted Berger Stigma Scale (5) | higher scores represent higher levels of internalized and anticipated stigma | [9-36]  17(13, 21)  α=0.74 |
| 13 | Social support network | Sum of 12 items adapted the Social Support Behaviors Scale at 48 months (6)(friends and parents/guardians) † | higher scores represent greater social support. | [21-60]  45 (39, 50)  α=0.68 |
| 14 | Distance to health facility | How far from your home is the hospital or health clinic at 48 months. | Very near/near=0; | 461 (72.4) |
|  |  |  | Very far/far/ don’t know/n/a/ missing =1 | 176 (27.6) |
|  | **ECONOMIC LEVEL FACTORS** | | | |
| 15 | Asset ownership | Family ownership of 20 selected assets at 36 months (7) | high possession [≥7 assets]=0 | 549 (86.2) |
|  |  |  | low possession [<7 assets]=1 | 88 (13.8) |
| 16 | Child poverty | Sum score of six items at 36 months(8) | Lower scores representing greater levels of poverty | [0-9]  4(3,5) |
| 17 | Economic Group assignment | Group assigned in RCT at baseline | Control=1 | 314 (49.3) |
|  |  |  | Intervention=2 | 323 (50.7) |

†social support network included from parents and friends only. Social support from classmates and teachers were not assessed since many participants were not in school at the time. α-Cronbach's alpha in the sample. Non-biological caregivers included brother-in-law, counsellor, brother-in-law, friend, guardian, stepfather, or stepmother.

**SENSITIVITY ANALYSES**

We performed a sensitivity analysis to evaluate whether including cases with incomplete data in the analysis would affect model results. There were a total of n=46 (6.6%) missing on the outcome, n=42 (5.9%) were missing adherence history, and n=40 were missing for age, substance use, depression, hopelessness, adherence self-efficacy, HIV disclosure, ART treatment supporter, family cohesion, HIV-related stigma, social support network. distance to health facility, and child poverty. We used the expectation-maximization (EM) algorithm (9, 10) to impute missing values and refitted the predictive model to the imputed data using the same 10-fold cross-validation lasso technique described in the main analysis. We compared the predictors retained, AUC, and calibration to the model run on complete cases.

Seven predictors were retained as important in this sensitivity analysis. Five (previous adherence, adherence self-efficacy, child poverty, family cohesion and economic group assignment) were the same in the model developed in complete cases only (Table S3). In addition to these five, age and primary caregiver were also retained. The model’s ability to discriminate between ALWHIV with and without poor adherence was in the acceptable range (AUC=70). However, there were some areas of miscalibration observed; test statistic=5.97; p-value=0.015 (Figure S1). The model predictions overestimated the true observed rate of poor adherence for predicted probabilities between 0.16 and 0.20 only at the 95% confidence level. All other predicted values were in agreement with actual observed rate of poor adherence in the ALWHIV sample, at the 95% and 99% confidence levels.

**Table S3. Unstandardized penalized regression coefficients of predictors retained in the lasso model to predict poor adherence**

|  | **PREDICTORS** | **All missing values imputed**  **(N=702)** |
| --- | --- | --- |
|  | **Intercept** | -0.8632724 |
|  | **DEMOGRAPHIC FACTORS** |  |
| **1** | Age group | 0.0096742 |
| **2** | **Gender** | x |
|  | **INDIVIDUAL LEVEL FACTORS** | |
|  | **Behavioral** |  |
| **3** | Substance use | x |
| **4** | History of ART adherence | 1.21364 |
|  | **Psychosocial** |  |
| **5** | Depression | x |
| **6** | Hopelessness | x |
| **7** | Adherence Self-efficacy | -0.0001293 |
| **8** | HIV disclosure | x |
|  | **HOUSEHOLD LEVEL FACTORS** | |
| **9** | ART Treatment Supporter | x |
| **10** | Family cohesion | 0.0018997 |
| **11** | Caregiver type | -0.0476692 |
|  | **COMMUNITY/STRUCTURAL LEVEL FACTORS** | |
| **12** | HIV-related stigma | x |
| **13** | Social support network | x |
| **14** | Distance to health facility | x |
|  | **ECONOMIC LEVEL FACTORS** | |
| **15** | Asset ownership | x |
| **16** | Child poverty | -0.0476692 |
| **17** | Economic intervention group assignment | -0.2378935 |
|  | **Total number of predictors retained from the 17 in the model** | 7 |
|  | **AUC (Bootstrap corrected 95% CI) derived using 10-fold cross-validation** | 70.2 (62.0, 71.3) |
|  | **AUC (95% CI) derived using 1000 bootstrap resampling which adjusted for clustering by clinics** | 70.1 (66.3, 73.7) |
|  | **Lambda** | 0.0184781 |

**'x'**-excluded from final prediction model after lasso regression. AUC=area under the curve. 95% CI=95% confidence interval. NB: penalized regression coefficients were derived after a penalty was applied which reduces overfitting of the data during model development. The penalized coefficients are not reflective of true population-level associations, since these are biased, and so attention should not be placed on interpreting individual predictor coefficients, but on how the model performs with the combination of all predictors together.

**Figure S1. Calibration belt showing deviations from the bisector (45% line of perfect fit) at the 95% (inner belt: light grey area) and 99% (outer belt: dark grey area) confidence levels for the model in which all missing values were imputed.**

**References**

1. Kovacs M. Children’s Depression Inventory. A measure of depressive symptoms in children and adolescents. North Tonawanda: Multi-Health Systems Inc; 1992.

2. Beck AT, Weissman A, Lester D, Trexler L. The measurement of pessimism: the hopelessness scale. Journal of consulting and clinical psychology. 1974;42(6):861-5.

3. Johnson MO, Neilands TB, Dilworth SE, Morin SF, Remien RH, Chesney MA. The role of self-efficacy in HIV treatment adherence: validation of the HIV Treatment Adherence Self-Efficacy Scale (HIV-ASES). Journal of behavioral medicine. 2007;30(5):359-70.

4. Moos RH. Family environment scale manual: Development, applications, research: Consulting Psychologists Press; 1994.

5. Berger BE, Ferrans CE, Lashley FR. Measuring stigma in people with HIV: Psychometric assessment of the HIV stigma scale¶. Research in nursing & health. 2001;24(6):518-29.

6. Vaux A, Riedel S, Stewart D. Modes of social support: The social support behaviors (SS‐B) scale. American Journal of Community Psychology. 1987;15(2):209-32.

7. Ssewamala FM, Han C-K, Neilands TB. Asset ownership and health and mental health functioning among AIDS-orphaned adolescents: Findings from a randomized clinical trial in rural Uganda. Social Science & Medicine. 2009;69(2):191-8.

8. Karimli L, Ssewamala FM, Neilands TB, Wells CR, Bermudez LG. Poverty, economic strengthening, and mental health among AIDS orphaned children in Uganda: Mediation model in a randomized clinical trial. Soc Sci Med. 2019;228:17-24.

9. Little RJ, Rubin DB. Statistical analysis with missing data: John Wiley & Sons; 2019.

10. Dempster AP, Laird NM, Rubin DB. Maximum Likelihood from Incomplete Data Via the EM Algorithm. Journal of the Royal Statistical Society: Series B (Methodological). 1977;39(1):1-22.
